# Ca^2+^/Calmodulin-Dependent Protein Kinase IV Promotes Interplay of Proteins in Chromatoid Body of Male Germ Cells

**DOI:** 10.1038/srep12126

**Published:** 2015-07-16

**Authors:** Guishuan Wang, Huijuan Zhang, Lu Wang, Yuan Wang, Hefeng Huang, Fei Sun

**Affiliations:** 1 International Peace Maternity & Child Health Hospital, Institute of Embryo-Fetal Original Adult Disease Shanghai Key laboratory for Reproductive Medicine, School of Medicine, Shanghai Jiaotong University, Shanghai 200030, China

## Abstract

The chromatoid body is a granule-like structure of male germ cells, containing many proteins and RNAs, and is important for spermatogenesis. However, the molecular mechanisms for the formation and function of the chromatoid body are still elusive. Here, we report that Ca^2+^/calmodulin-dependent protein kinase IV (CaMKIV) accumulates in the chromatoid body by immunofluorescence staining, indicating that CaMKIV is a new component of the chromatoid body. Furthermore, we find that CaMKIV can interplay with the other components of the chromatoid body by immunoprecipitation: mouse VASA homologue (MVH), mouse homologue of PIWI, PIWIL1 (MIWI), and kinesin KIF17b. Importantly, interplay between KIF17b and MVH or MIWI can be potentially regulated by CaMKIV. These results imply that CaMKIV plays a role in maintenance the structure of chromatoid body by regulating the associations of proteins in it.

The chromatoid body was first described by Benda in 1891^1^ and, since then, it has attracted the interest of many researchers. Chromatoid bodies can be detected in the cytoplasm of meiotic spermatocytes and are characterized as fibrous-granular structures that are formed between mitochondria clusters^2^. After meiosis, a mature chromatoid body appears, and the fibrous-granular structure is compacted into a finely filamentous and lobulated granule, bouncing around at the surface of the nucleus of round spermatid^2^,^3^. This structure remains in the cytoplasm of the spermatid until the nucleus begins to elongate and finally disappears late in spermiogenesis^2^,^4^.

Chromatoid bodies contain many RNA-binding proteins and RNA strands^5^,^6^, and on the basis of its structural features and composition, it is considered as a specialized form of germplasm or nuage^7^. Thus, chromatoid bodies are proposed as RNA-processing centers of male germ cells^5^. Mouse VASA homologue (MVH), a DEAD-box RNA helicase, localizes in the chromatoid body^8^,^9^ and regulates RNA granules^10^. Another RNA-binding protein, mouse homologue of PIWI, PIWIL1 (MIWI) also localizes in the chromatoid body^8^ and physically interacts with MVH^11^. MIWI is an important component of the chromatoid body, because *Miwi*-null mice lack the normal structural features of a chromatoid body, and the fibrous-granular structure fails to condense into the mature chromatoid body^12^. Chromatoid bodies also contain many non-RNA-binding proteins, and kinesin KIF17b is one of them. KIF17b has been shown to regulate spermatids transcription activity by shuttling between the nucleus and the cytoplasm^13^,^14^. In the cytoplasm of spermatids, KIF17b accumulates in the chromatoid body and associates with MIWI, thus KIF17b may be related to the movement of the chromatoid body^12^.

Ca^2+^/calmodulin-dependent protein kinase IV (CaMKIV) is expressed in the testes, brain, thymus and other tissues^15^. In mouse testes, CaMKIV is expressed in spermatogonia and spermatids but not spermatocytes^16^, and spermiogenesis is impaired in mice lacking CaMKIV^17^. Here we report that CaMKIV is localized in the chromatoid body and interacts with the components of the chromatoid body: MVH, MIWI and KIF17b. Both MVH and MIWI bind to the motif of Arg–Lys–Lys–Ser at KIF17b, which is also the substrate recognition motif of CaMKIV^18^. And more importantly, CaMKIV can regulate the interplay of KIF17b between MVH and MIWI. These results indicate that CaMKIV is a new component of the chromatoid body and plays an important role in maintaining the structure of chromatoid body through regulating interactions of proteins.

## Results

### CaMKIV is localized in the chromatoid body

**Figure 1:**
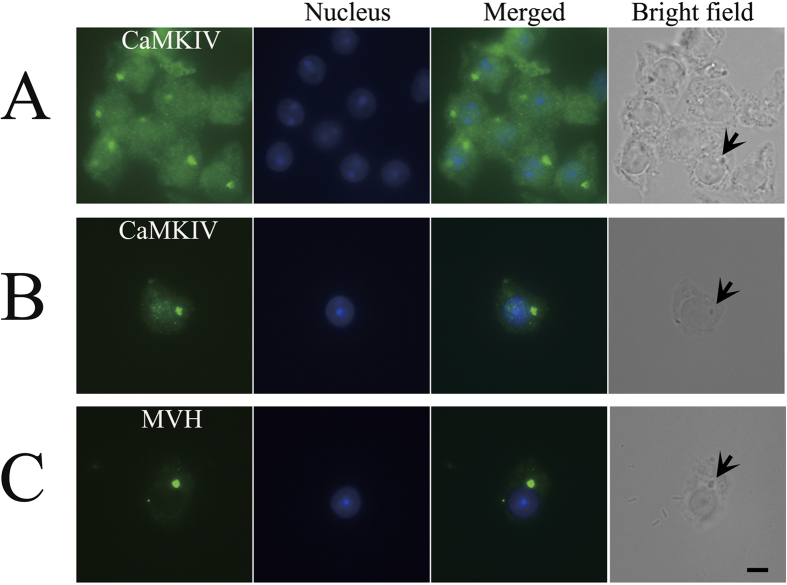
Localization of CaMKIV in the chromatoid body. Testes of adult mice were subjected to squash preparation and then immunofluorescence staining. (**A,B**) The round spermatids were immunostained with anti-CaMKIV antibody (green). (**C**) Immunofluorescence staining of MVH (green). The parallel bright-field images demonstrate the location of the chromatoid bodies, which are indicated by arrows. Alexa Fluor 488 anti-rabbit IgG was used as a secondary antibody, and nuclei were stained blue with Hoechst 33342 dye. Scale bar: 5 μm.

It has been reported that CaMKIV shuttles between the nuclear and cytoplasmic compartments of RAW 264.7 cells^19^, which indicates that CaMKIV might be localized in the cytoplasmic compartments in germ cells, such as the localization of KIF17b in mouse testes^20^. Immunofluorescence was carried out on squash preparations to study the localization of CaMKIV in great detail. Interestingly, we found that, in the cytoplasm of spermatids, the signal condensed into one area (Fig. 1A,B) and overlapped with the phase-contrast image of the chromatoid body (Fig. 1A,B). MVH antibody was used as a positive control for chromatoid-body staining (Fig. 1C). These results show that CaMKIV is a new component of the chromatoid body.

### CaMKIV interacts with the MVH and MIWI, components of the chromatoid body

**Figure 2:**
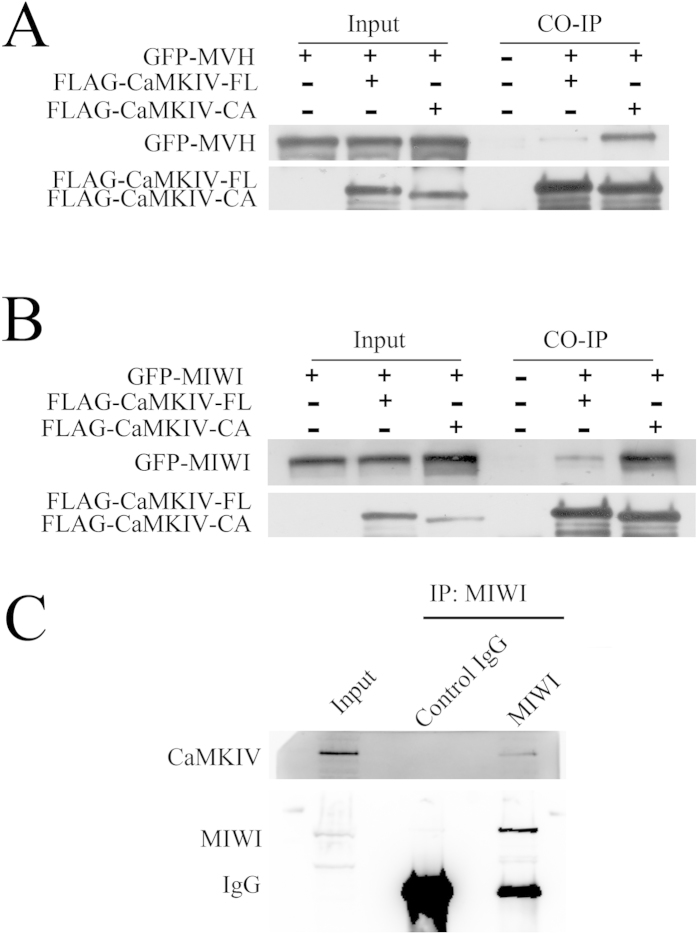
Interaction of CaMKIV with MVH and MIWI. (**A**) In HEK293T cells, plasmids of FLAG-CaMKIV-FL (the full length of CaMKIV) or FLAG-CaMKIV-CA (constitutively active form of CaMKIV) were co-transfected with GFP-MVH; (**B**) plasmids of FLAG-CaMKIV-FL or FLAG-CaMKIV-CA were transfected with GFP-MIWI. Immunoprecipitation from the cell lysates was performed with anti-mouse FLAG antibody, and the samples were immunoblotted by anti-mouse GFP antibody to detect MVH or MIWI, and then by anti-mouse FLAG antibody to detect coimmunoprecipitated CaMKIV. (**C**) Mouse testes were immunoprecipitated by anti-rabbit MIWI antibody, and the samples were immunoblotted by anti-rabbit MIWI antibody, then by anti-mouse CaMKIV antibody to detect coimmunoprecipitated CaMKIV. Anti-rabbit GFP antibody was used as a negative control.

As CaMKIV is a member of the chromatoid body, we guess that CaMKIV may interplay with MVH and MIWI. To confirm this, co-immunoprecipitation experiments were carried out after the co-expression of FLAG-CaMKIV-FL (the full length of CaMKIV) or FLAG-CaMKIV-CA (the active form of CaMKIV) with GFP-MVH or GFP-MIWI in HEK293T cells. FLAG-CaMKIV-FL and FLAG-CaMKIV-CA were immunoprecipitated using FLAG antibody, immunoblotting with GFP antibody revealed that GFP-MVH and GFP-MIWI were co-precipitated with FLAG-CaMKIV-FL, and the association was greatly increased when FLAG-CaMKIV-CA was co-expressed with GFP-MVH or GFP-MIWI (Fig. 2A,B). Mouse testes were immunoprecipitated by MIWI antibody, immunoblotting with CaMKIV antibody showed that CaMKIV associated with MIWI (Fig. 2C). These data show that CaMKIV is a new member of the chromatoid body and associates with MVH and MIWI.

### CaMKIV interplays with another component of the chromatoid body: KIF17b

**Figure 3:**
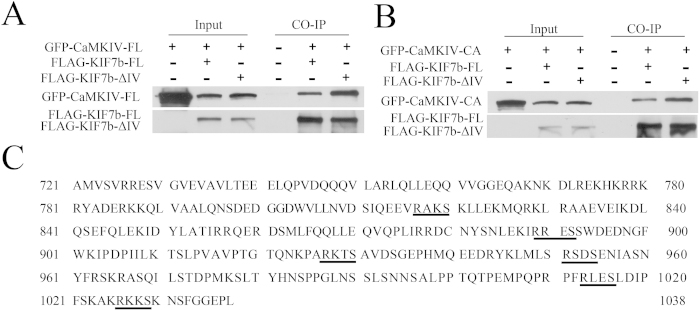
Interaction of CaMKIV with KIF17b. Plasmids encoding the full length of KIF17b (FLAG-KIF17b-FL) or the mutant form of KIF17b with R-K-K-S deletion (FLAG-KIF17b-ΔIV) were co-expressed with (**A**) GFP-CaMKIV-FL or (**B**) GFP-CaMKIV-CA in HEK293T cells. The cell lysates were incubated with anti-mouse FLAG antibody and CaMKIV was immunoblotted by anti-mouse GFP antibody, whereas KIF17b was detected by anti-mouse FLAG antibody, (**C**) The R-X-X-S/T motifs at the CTD of KIF17b are underlined.

The R-K-K-S sequence is the substrate recognition motif of CaMKII/CaMKIV^18^, and CaMKII can bind to the Arg-Lys-Lys-Ser (R-K-K-S) sequence of KIF17 in the mouse brain^21^. Therefore, it is possible that CaMKIV may associate with KIF17b, both of which are components of the chromatoid body. In addition, the Germ Online database was used to analyze the expression of CaMKII in the testes^22^ and found that CaMKII had a very low expression level in spermatids; therefore, we focused on CaMKIV in this study. As the R-K-K-S sequence is the substrate-recognition motif of CaMKIV, the mutant form of KIF17b (FLAG-KIF17b-ΔIV) with R-K-K-S deletion or the full length of KIF17b (FLAG-KIF17b-FL) was co-transfected with GFP-CaMKIV-FL or GFP-CaMKIV-CA in HEK293T cells. The cell lysate was incubated with the FLAG antibody. Immunoblotting with GFP antibody revealed that FLAG-KIF17b-FL co-precipitated with GFP-CaMKIV-FL and GFP-CaMKIV-CA (Fig. 3A,B). However, the deletion of the R-K-K-S sequence did not reduce the association of KIF17b with CaMKIV (Fig. 3A,B). We analyzed the amino-acid sequence of KIF17b and found that there were multiple R-X-X-S/T motifs at the C-terminal domain (CTD) of KIF17b (Fig. 3C), which were potential binding sites for CaMKIV^18^; therefore, the deletion of the R-K-K-S sequence did not decrease the interaction of KIF17b with CaMKIV. These data show that KIF17b has multiple binding sites for CaMKIV.

### MVH and MIWI associate with the tail of KIF17b

**Figure 4:**
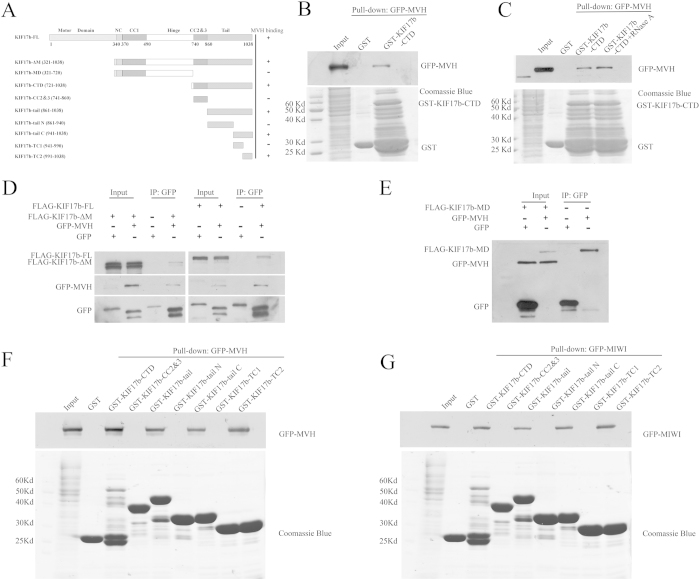
Interaction of MVH and MIWI with the tail of KIF17b. (**A**) Schematic diagram of the full length and truncations of KIF17b; the residue numbers at the domain boundaries are indicated. (**B,C**) The interaction of KIF17b-CTD with MVH was assayed by the GST pull-down assay. Purified GST-KIF17b was used to absorb GFP-MVH from the lysates of HEK293T cells. The bound materials were then subjected to SDS-PAGE and immunoblotted with anti-GFP antibody or treated with RNase A before SDS-PAGE. The GST-fusion proteins were shown by Coomassie brilliant blue R-250 staining of the gels. (**D**) Immunoprecipitation of KIF17b by MVH. Expression plasmid of the full length of FLAG-tagged KIF17b, or plasmid of the motor-domain-deleted form of FLAG-tagged KIF17b (FLAG-KIF17b-ΔM) and GFP-tagged MVH were co-transfected to HEK293T cells, and the cell lysates were precipitated by the anti-rabbit GFP antibody, and immunoblotted with anti-mouse GFP antibody or anti-mouse FLAG antibody. (**E**) Plasmids of the middle domain of KIF17b (FLAG-KIF17b-MD) and GFP-MVH were transfected into HEK293T cells and then the cell lysates were immunoprecipitated by the GFP antibody. (**F,G**) Mapping of the MVH and MIWI binding sites of KIF17b-CTD was completed using the GST pull-down assay. Purified truncations of GST-KIF17b-CTD were used to precipitate GFP-MVH or GFP-MIWI from the lysates of HEK293T cells. The precipitates were then subjected to SDS-PAGE and immunoblotted with anti-GFP antibody; the gels were then stained by Coomassie brilliant blue R-250.

It has been reported that the tail of KIF17 had a cargo binding site and that the cargo could be released by controlling CaMKII^21^. Therefore, we assumed that the tail of KIF17b had some cargo in which the interactions with KIF17b could be regulated by CaMKIV. To confirm this, a GST pull-down assay was performed to identify the cargoes of KIF17b in testis using the CTD of KIF17b as bait. The truncations of KIF17b are shown in Fig. 4A. A new cargo of KIF17b was identified by 1D LC-MS. The new cargo is MVH, which is also a component of the chromatoid body^23^. The association was confirmed by the GST pull-down assay (Fig. 4B), and RNase A treatment showed that the association was RNA-independent (Fig. 4C). The interaction of KIF17b with MVH was confirmed by co-immunoprecipitation experiment (Fig. 4D). The motor domain of KIF17b was not necessary for MVH binding at, since the mutant form of KIF17b with deletion of the motor domain (FLAG-KIF17b-ΔM) was still immunoprecipited by GFP-MVH (Fig. 4D), but the middle domain of KIF17b (KIF17b-MD) did not have MVH binding activity (Fig. 4E). These data indicate that MVH mainly associates with the CTD of KIF17b. Then, we mapped the interactions between MVH and KIF17b CTD using constructs that encoded the truncated KIF17b (KIF17b-CC2&3, KIF17b-tail, KIF17b-tail N, KIF17b-tail C, KIF17b-TC1, and KIF17b-TC2) with the GST tag. Purified GST-fusion of KIF17b truncations were used to pull-down 293T cell lysates that were transfected with GFP-MVH and the truncations of KIF17b-tail, KIF17b-tail C, and KIF17b-TC2 associated with MVH (Fig. 4F). As MIWI also interplays with KIF17b^14^, the GST pull-down assay was performed in order to test the interaction of MIWI with KIF17b by GSTKIF17b-CTD and found that MIWI could interact with the CTD of KIF17b (Fig. 4G). While using the CTD truncations of KIF17b, GST-pull down assay found that KIF17b-tail C and KIF17b-TC2 were associated with MIWI (Fig. 4G), which is the same as MVH. The results of the GST pull-down assay show that the 991–1038 amino acids of KIF17b (KIF17b-TC2) play an important role in the association of MVH, MIWI, and KIF17b. These data indicate that MVH and MIWI are both the cargo of KIF17b and that they both bind to the 991–1038 amino acids of the KIF17b tail.

### The sequence of R-K-K-S is the binding site of MVH and MIWI at KIF17b

**Figure 5:**
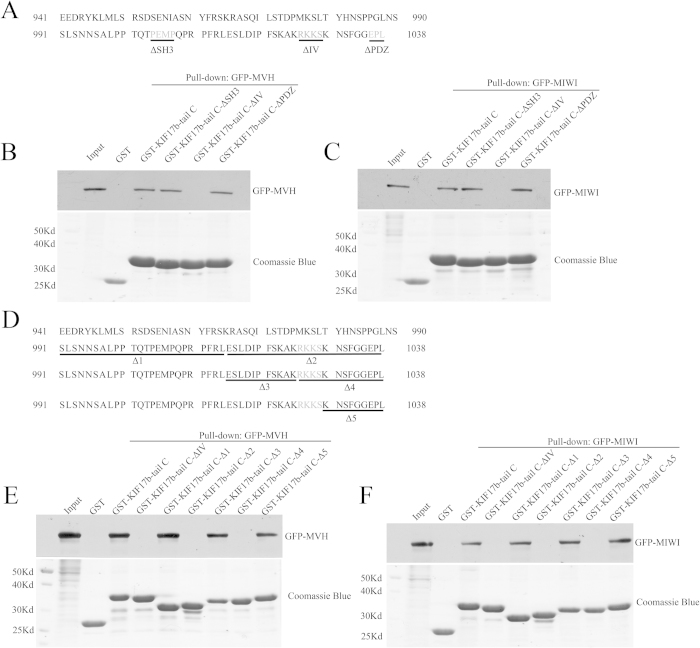
The sequence of R-K-K-S is the binding site of MVH and MIWI at KIF17b. (**A**) Schematic diagram of the mutations of KIF17b-tail C; the SH3, CaMKII/IV, and PDZ binding sites are underlined and gray. (**B**,**C**) Mapping of the MVH and MIWI binding sites of KIF17b-tail C, completed using the GST pull-down assay. (**D**) Schematic diagram of the mutations of KIF17b-tail C; the mutated sequence is underlined and the CaMKII/IV binding sites are gray. (**E,F**) The GST pull-down assay was used to certify the binding sites of MVH and MIWI at KIF17b-tail C.

Previous research has shown that KIF17b-TC2 contained the SH3, CaMKII/IV, and PDZ binding sites^21^. To identify the exact binding site, GST-fusion of KIF17b-tail C with deletion of the SH3 binding site (GST-KIF17b-tail C-ΔSH3), CaMKIV binding site (GST-KIF17b-tail C-ΔIV), or PDZ binding site (GST-KIF17b-tail C-ΔPZD) (Fig. 5A) were used to pull-down HEK293T cell lysate transfected with GFP-MVH or GFP-MWI. We found that the deletion of the CaMKIV binding site abolished the association of MVH and MIWI between KIF17b, whereas deletion of the SH3 and PDZ binding sites did not affect the association (Fig. 5B,C). Furthermore, truncations of KIF17b-tail C (Fig. 5D) were used to perform the GST pull-down assay, and only the R-K-K-S sequence deletion affected the association of MVH and MIWI between KIF17b (Fig. 5E,F). These data show that MVH and MIWI both bind to the R-K-K-S motif of KIF17b.

### CaMKIV stimulates the association of MVH and MIWI between KIF17b

**Figure 6:**
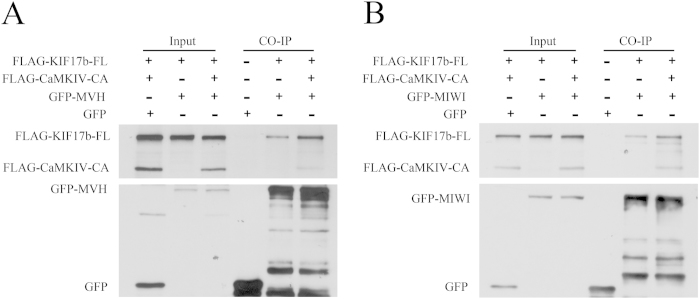
CaMKIV stimulates the association of MVH and MIWI with KIF17b. HEK293T cells were transfected with the indicated combinations of expression plasmids for FLAG-KIF17b-FL, GFP-CaMKIV-CA, GFP-MVH, and GFP-MIWI. The cell lysates were precipitated by anti-mouse GFP antibody. KIF17b and CaMKIV were immunoblotted by anti-mouse FLAG antibody, whereas MVH and MIWI were immunoblotted by anti-mouse GFP antibody. The total DNA in each group was kept constant using an empty FLAG plasmid.

**Figure 7:**
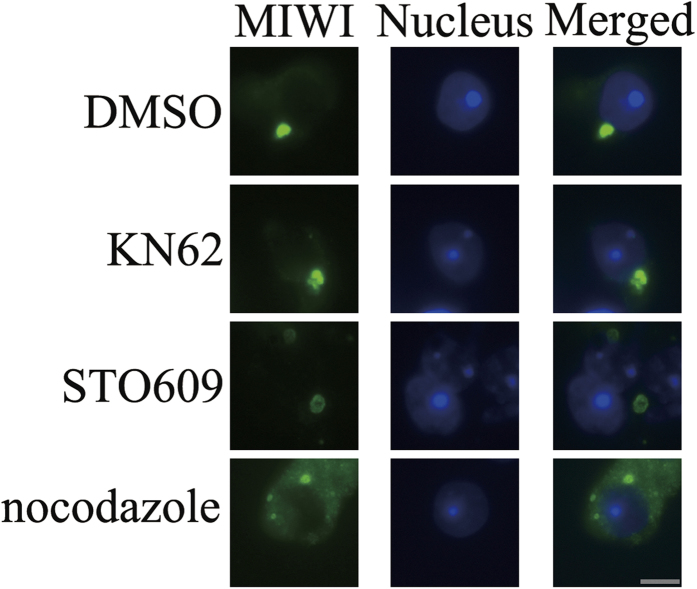
Inhibitors of CaMKIV affect the structure of chromatoid body. Seminiferous tubules were treated with with 25 μl/ml DMSO, 60 μM KN62, 10 μM STO609, 20 μg/ml nocodazole for 48 h. DMSO was used as a negative control; CaMKIV inhibitors, KN62 and STO609, were used to treat the samples; microtubule inhibitor, nocodazole, was used as a positive control. After incubation, tubules were performed to immunofluorescence by squash preparation, the chromatoid body was stained by MIWI, Alexa Fluor 488 anti-rabbit IgG was used as a secondary antibody, and nuclei were stained blue with Hoechst 33342 dye. Scale bar: 5 μm.

It has been reported that cargo-release from KIF17 was regulated by CaMKII in mouse brain^21^. Therefore, it is important to find out whether CaMKIV can affect the association of MVH and MIWI between KIF17b. To confirm this, a co-immunoprecipitation experiment was carried out after co-expression of FLAGKIF17b-FL, FLAG-CaMKIV-CA, and GFPMVH or GFP-MIWI in HEK293T cells. The cell lysate was incubated with GFP antibody. Immunoblotting with FLAG antibody revealed that the FLAG-CaMKIV significantly stimulated the association between FLAG-KIF17b-FL and GFP-MVH, as well as FLAG-KIF17b-FL and GFP-MIWI (Fig. 6A,B). To confirm these results, seminiferous tubules were incubated for 48 h with microtubule inhibitor, nocodazole^24^ and CaMKIV inhibitors, KN62 and STO609^19^,^25^,^26^. Then samples were subjected to immunofluorescence by squash preparation and the chromatoid body was indicated by staining of MIWI. As showed in Fig. 7, chromatoid body was less compacted after incubation with KN62 or STO609, while it disintegrated to form several small spheres after incubation with nocodazole as reported before^24^. These data indicate that CaMKIV maintains the compact structure of chromatoid body by promoting the associations of MVH and MIWI with KIF17b.

## Discussion

Spermatogenesis is a dynamic and well organized process and is supported by Sertoli cells^27^. This process is regulated by specialized genetic and epigenetic pathways of gene regulation^28^. During the late steps of spermatogenesis, transcription of the haploid genome is silenced by the compaction of the haploid genome through histone-to-protamine transition^29^,^30^. However, during these steps, the protein synthesis of a large number of specific genes is still ongoing, which are required for the last steps of sperm development^31^. Thus, mRNA storage and processing are crucial during these steps and, interestingly, many mRNA binding proteins have been identified in male germ cells, regulating the stability and translation of target mRNA strands^32^,^33^.

Recent research findings support the hypothesis that the chromatoid body serves as an RNA processing center for male germ cells, based on its structural features and composition^5^,^10^,^34^,^35^. The chromatoid body contains many RNA-binding proteins, and these proteins form a complicated and dynamic complex through protein interactions^12^,^35^,^36^. However, the mechanism that regulates the formation of the chromatoid body remains unclear. High levels of arginine methylation have been reported in the chromatoid body, and one of the well-known components, MIWI, was arginine-methylated at the *N* terminus^37^; the arginine methyl marks could be read by a family of Tudor domain proteins^38^. The interaction of MIWI with Tudor-domain proteins, mediated by arginine methylation, is crucial for the cytoplasmic granular localization of MIWI and the formation of the chromatoid body in round spermatids^39^,^40^,^41^.

It has been reported that CaMKIV is localized in the nucleus of spermatids^16^ and that it plays important roles in the histone-to-protamine transition, and spermiogenesis is impaired in mice lacking CaMKIV^17^. However, Chatila lab finds that CaMKIV-deficient male mice were fertile and did not affect spermatogenesis^42^. This discrepancy may come from the different gene-targeting strategies. Moreover, just like KIF17b in mouse testes^20^, CaMKIV has the ability to shuttle between the nucleus and cytoplasm^19^,^43^. Therefore, the localization of CaMKIV in mouse testes was studied in great detail. Herein, we report that CaMKIV was localized in the chromatoid body and was a new component of the chromatoid body (Fig. 1). This result reveals that CaMKIV not only plays a role in the nucleus, but also has crucial functions in the cytoplasm of spermatids.

To validate the fact that CaMKIV is a component of the chromatoid body, immunoprecipitation experiments were used to detect whether CaMKIV interacts with MVH and MIWI, which are two well-studied components of the chromatoid body. The experimental results showed that CaMKIV associated with MVH and MIWI; moreover, the constitutively active form of CaMKIV had a stronger interaction with MVH than MIWI (Fig. 2). These results indicate that CaMKIV may function through the active form in the chromatoid body. More interestingly, in mouse brain, CaMKII interacts with KIF17 at the R-K-K-S sequence and regulates the cargo release from KIF17^21^. CaMKII and CaMKIV have some similar characteristics, such as both of them recognize the motif of R-X-X-S/T, in most cases^18^. The interaction of CaMKIV with KIF17b was validated by immunoprecipitation experiments and found that the R-K-K-S deletion did not decrease the interaction (Fig. 3A,B). This is possibly due to the fact that there are multiple R-X-X-S/T motifs, that is, the substrate recognition motif of CaMKIV, in the C-terminal domain of KIF17b (Fig. 3C).

GST pull-down experiments were performed to identify the cargoes of KIF17b at the C-terminal domain, and MVH was found as a new cargo (Fig. 4). Through truncations of KIF17b, the MVH binding site was restricted to the 991–1038 amino acids of KIF17b (Fig. 4). In addition, by the mutation assay, the binding site was mapped at the R-K-K-S motif (Fig. 5). It has been reported that MIWI could interact with KIF17b in the chromatoid body^12^, and GST pull-down experiments showed that MIWI could interact with the tail of KIF17b (Fig. 4). Interestingly, the binding site of MIWI was also mapped at the R-K-K-S motif (Fig. 5). As KIF17 is homodimeric and very similar to KIF17b^44^, it is possible that, in the chromatoid body, the heterodimer of MVH and MIWI^45^ interacts with the homodimer of KIF17b to form a heterotetramer. Importantly, the heterotetramer is regulated by CaMKIV, as the results of immunoprecipitation showed that the interaction of KIF17b with MVH and MIWI was enhanced by CaMKIV (Fig. 6); this is a different regulation model compared with CaMKII^21^. The structure of chromatoid body is disrupted after incubation with inhibitors of CaMKIV (Fig. 7), indicating CaMKIV takes part in the structure maintenance of chromatoid body.

After the analysis of the amino-acid sequence, many proteins in the chromatoid body were found to contain the R-X-X-S/T motif, such as MVH, MIWI, Dcp1a, GW182, and the Tudor-domain proteins. This indicates that CaMKIV may regulate the chromatoid body in a basal and general way, such as the regulation of the interaction of KIF17b with MVH and MIWI. CaMKIV not only plays a crucial role in chromatin compaction in the nucleus of spermatids during the late stages of spermatogenesis, but it also regulates the germ-cell-specific RNA-processing center in the chromatoid body, that is, in the cytoplasm of round spermatids preceding the histone-to-protamine transition and transcriptional silencing.

## Methods

### Ethics statement

All animal care and experiments of this study were performed in accordance with the guidelines and were approved by the Ethics Committee of International Peace Maternity & Child Health Hospital, School of Medicine, Shanghai Jiaotong University.

### Animals

C57BL/6 male mice were originally purchased from Vital River Laboratories in Beijing, China. The mice were kept at temperatures of 22 °C with light cycles of 14 h light and 10 h dark; they were provided food and water ad libitum.

### Cell culture and transfection

HEK293T cells were cultured in Dulbecco’s modified Eagle’s medium (DMEM, Invitrogen) that was supplemented with 10% fetal bovine serum (FBS, Invitrogen) and 1% antibiotics (100 U/mL penicillin and 100 μg/mL streptomycin, Invitrogen), and were cultured at 37 °C with 5% CO_2_. Cells were transfected by the Lipofectamine 2000 reagent (Invitrogen). The transfection procedure was performed according to the manufacturer’s instructions.

### Plasmid construction

The full-length mice KIF17b, MVH, MIWI and CaMKIV were amplified by the polymerase chain reaction (PCR) in cells from mouse testes using the primers containing specific restriction sites. To construct the expression vectors, KIF17b was cloned into p3 × FLAG-*myc*-CMV-24 (Sigma), MIWI into pEGFP-C1 (Clontech), and MVH and CaMKIV into p3xFLAG-*myc*-CMV-24 and pEGFP-C1, respectively. The following primers were used: KIF17b-FL (aa 1-1038), forward, that is, 5′-TAATGAATTCCATGGCCTCGGAGTCAGTGA-3′, and reverse, 5′-AATAGTCGACATCACAGAGGCTCACCACCG-3′. MVH: forward 5′-GCGCAGATCTAGCTATCATGGGAGATGAAG-3′ and reverse 5′-GCTAGTCGACGCTTTAATCCCATGACTCGT-3′. MIWI: forward, 5′-TATAGAATTCAATGACTGGCCGAGCCCGAG-3′ and reverse 5′-TATAGTCGACGTTAGAGGTAGTAGAGGCGG-3′. Full-length primer CaMKIV (GFP-CaMKIV-FL): forward 5′-ATATGGTACCATGCTCAAAGTCACGGTGCCC-3′ and reverse 5′-ATATTCTAGAGTACTCTGGCTGAATCGCAT-3′. FLAG-CaMKIV-FL: forward 5′-ATATGGTACCGATGCTCAAAGTCACGGTGCC-3′ and reverse 5′-ATATTCTAGAGTACTCTGGCTGAATCGCAT-3′. The active form of CaMKIV (CaMKIV-CA) (Δaa FNARRKLK): forward 5′-CAGAAGAAACTTCAAGAGGCAGCGGTGAAG-3′ and reverse 5′-CTTCACCGCTGCCTCTTGAAGTTTCTTCTG-3′. The truncations of KIF17b were generated by PCR and then subcloned into pGEX-5X-3 (GE Healthcare) and p3 × FLAG-*myc*-CMV-24 expression vector.

### Western blotting

Tissues and cells were lysed in RIPA buffer (50 mM Tris-HCl, pH 7.4, 150 mM NaCl, 1% Triton X-100, 1% sodium dodecyl sulfate, 1% sodium deoxycholate, 1 mM EDTA) containing a completely EDTA-free protease inhibitor cocktail (Roche), 1 mM phenylmethylsulfonyl fluoride (PMSF) and phosphatase inhibitors (5 mM sodium orthovanadate). Protein lysates were loaded on SDS-PAGE gels and electroblotted onto nitrocellulose membranes (Amersham Biosciences). The nitrocellulose membranes were blocked for 1 h in 5% nonfat milk in TBST (10 mM Tris, pH 7.5, 200 mM NaCl, and 0.2% Tween 20) followed by incubation with primary antibodies. Mouse anti-GFP antibody (Clontech), rabbit anti-GFP antibody (Clontech), mouse anti-FLAG antibody (Sigma), rabbit anti-FLAG antibody (Sigma) and mouse anti-CaMKIV (Abnova) were used, and the bound antibodies were visualized by Lumi-Phos WB Chemiluminescent Substrate (Thermo Scientific).

### GST-pull-down assay

The GST-fusion of KIF17b truncations were expressed in the BL21 strain of *Escherichia coli* at 28 °C and purified by glutathione-Sepharose 4B beads (GE healthcare). The purified GST-fusion proteins were bound to the Sepharose beads and then incubated with testes lysates or 293 T cell lysates transfected with the indicated plasmids. The GST precipitates were subjected to RNase A (Takara) treatment at 37 °C for 1 min, or directly to SDS–PAGE, and transferred onto nitrocellulose membranes, or the gels were stained with 0.25% Coomassie brilliant blue R-250.

### Immunoprecipitation assay

The cells or mice testes were lysed in TNE buffer (10 mM Tris-HCl pH 7.5, 150 mM NaCl, 1 mM EDTA and 1% Nonidet P-40) with protease inhibitor cocktail, 1 mM PMSF and 5 mM sodium orthovanadate. The lysates were centrifuged at 18,000 × *g* for 10 min at 4 °C, and the supernatants were subjected to preclearing with Protein G Sepharose 4 fast flow (GE Healthcare) for 2 h at 4 °C on a turning wheel. After preclearing, the lysates were immunoprecipitated with the anti-FLAG, anti-GFP or anti-MIWI (Cell Signaling Technology) antibodies overnight at 4 °C on a turning wheel. The immunoprecipitates were separated by SDS-PAGE and transferred onto nitrocellulose membranes.

### Immunofluorescence

Squash samples were prepared as previously described^46^. The testes of adult C57Bl/6 mice were dissected and fixed in freshly prepared 2% formaldehyde in PBS (phosphate-buffered saline) (137 mM NaCl, 2.7 mM KCl, 10.1 mM Na_2_HPO_4_, 1.7 mM KH_2_PO_4_ pH 7.4) containing 0.05% Triton X-100 (Sigma) and 5 mM sodium orthovanadate for 5–10 min at room temperature. During this time, seminiferous tubules were liberated and dispersed in the fixative. Pieces of tubules were placed on a slide and gently minced with tweezers, and then a coverslip was added and the cells were squashed by exerting pressure on the coverslip. The slides were frozen in liquid nitrogen and the coverslips were removed. The slides were immediately placed in PBS for three subsequent 5 min rinses and blocked 1 h in 5% BSA in PBS. The primary antibody incubation was carried out at 4 °C in 1% BSA solution with CaMKIV antibody (Abcam) and MVH antibody (Abcam). Alexa Fluor 488 donkey anti-rabbit immunoglobulin G (IgG) (Molecular Probes) was used as secondary antibody. The nuclei were stained by Hoechst 33342 (Sigma) and the slides were digitally imaged using a fluorescence microscope (Nikon, T80i, Japan).

### Inhibition studies with nocodazole, KN62 and STO609

The drugs were dissolved in dimethyl sulfoxide (DMSO, Sigma). Seminiferous tubules were transferred to 96-well plate containing either DMEM alone supplemented with 25 μl/ml DMSO and 20 μg/ml nocodazole (Sigma), 60 μM KN62 (Sigma) and 10 μM STO609 (Sigma). The tubules were incubated for 48 h at 34 °C in an atmosphere containing 5% CO_2_ in air. After incubation, tubules were performed to immunofluorescence by squash preparation.

## Additional Information

**How to cite this article**: Wang, G. *et al.* Ca2^+^/Calmodulin-Dependent Protein Kinase IV Promotes Interplay of Proteins in Chromatoid Body of Male Germ Cells. *Sci. Rep.* **5**, 12126; doi: 10.1038/srep12126 (2015).
